# The Strain Response to Intraocular Pressure Increase in the Lamina Cribrosa of Control Subjects and Glaucoma Patients

**DOI:** 10.1167/tvst.13.12.7

**Published:** 2024-12-04

**Authors:** Cameron A. Czerpak, Michael Saheb Kashaf, Brandon K. Zimmerman, Rebecca Mirville, Nicolas C. Gasquet, Harry A. Quigley, Thao D. Nguyen

**Affiliations:** 1 Department of Mechanical Engineering, The Johns Hopkins University, Baltimore, MD, USA; 2 Wilmer Ophthalmological Institute, Department of Ophthalmology, Johns Hopkins University School of Medicine, Baltimore, MD, USA; 3 Meharry Medical College, Nashville, TN, USA

**Keywords:** glaucoma, lamina cribrosa (LC), optic nerve head (ONH), strain, intraocular pressure (IOP), digital volume correlation

## Abstract

**Purpose:**

The purpose of this study was to measure biomechanical strains in the lamina cribrosa (LC) of living human eyes undergoing intraocular pressure (IOP) increase.

**Methods:**

Healthy control subjects and patients with glaucoma underwent optical coherence tomographic (OCT) imaging of the LC before and after wearing of swim goggles that increased IOP (57 image pairs, 39 persons). Digital volume correlation was used to measure biomechanical strains in optic nerve head tissue and change in depth of the anterior border of the LC.

**Results:**

The mean IOP increase in both glaucoma and control eyes was 7.1 millimeters of mercury (mm Hg) after application of the goggles. Among glaucoma eyes, strains that were significant were: contractile *E_zz_* (average = −0.33%, *P* = 0.0005), contractile *E_θθ_* (average = −0.23%, *P* = 0.03), *E_max_* (average = 0.83%, *P* < 0.0001), and *Γ_max_* (average = 0.95%, *P* < 0.0001), whereas the average anterior LC depth (ALD) decreased by 2.39 µm (anterior; *P* = 0.0002). In glaucoma eyes, shear strain *E_z__θ_* was greater with worse mean deviation (MD) and visual function index (*P* = 0.044 and *P* = 0.006, respectively, multivariate models). Strain compliance for *E_r__θ_*, *E_z__θ_*, and *E_θθ_* all increased with greater MD worsening prior to imaging (*P* = 0.04, *P* = 0.007, and *P* = 0.03).

**Conclusions:**

LC strains were measurable 20 minutes after IOP increase, producing axial compression and greater peripheral strain than centrally. Some strain compliances were greater with worse existing visual field loss or with more progressive past field loss.

**Translational Relevance:**

Biomechanical strains are related to measures of glaucoma damage, supporting the hypothesis that optic nerve head biomechanical responses represent a noninvasive biomarker for glaucoma.

## Introduction

The mechanical behavior of tissues in the optic nerve head (ONH) region is an important element in its response to IOP,^1^ contributing to axon injury at an important site of damage.^2^ Although histological studies demonstrate the static state of these tissues, it is their viscoelastic behavior in the short term and their remodeling over time that can more directly inform how glaucoma damage occurs and how therapies to prevent injury might be developed. The behavior of the ONH and the lamina cribrosa (LC) have been modeled using ranges of tissue parameters, suggesting that the peripapillary sclera (PPS) is more resistant to strain than the LC, but that the two zones act as a unit.^3^^–^^6^ The biomechanical behavior of the human LC and sclera has been estimated in post mortem human control and glaucoma eyes.^7^^–^^11^ In such eyes, digital image correlation methods were applied to laser scanning microscopic images to derive the stress-strain relationships of the LC connective tissue.^12^^–^^16^ Midgett et al.^17^ showed that the resolution of the laser scanning microscopy in the scanning direction prevented accurate calculation of the anterior-posterior strain, indicating the local compression/tension in the thickness direction and the shear strains in the longitudinal planes of the peripapillary sclera and lamina cribrosa. However, the biomechanical response measured in the ex vivo inflation tests is not representative of the in vivo strain response because the eye has been removed from its physiological support and loading conditions, which includes the retrolaminar pressure and the extraocular tissues resisting the posterior movement of the eye wall in response to an intraocular pressure (IOP) increase.

To study LC tissue responses in vivo, the position of the anterior border of the LC and estimated strains within the LC have been measured in images from spectral domain—optical coherence tomography (OCT) obtained at two different intraocular pressures (IOPs),^18^^–^^23^ most recently in human brain-dead donors.^24^ Girard et al.^25^ and Nguyen and co-workers^17^ combined such OCT images of the LC and digital volume correlation (DVC) image analysis to calculate the LC strain response shortly after IOP elevation or decrease.^26^^,^^27^ In patients with open angle glaucoma (OAG),^28^ LC strains with short-term IOP change were greater in eyes with worse visual field sensitivity.^29^^–^^31^ Using suture lysis after trabeculectomy and images taken before and 20 minutes after IOP-lowering,^29^ we found that the short-term strain responses of the LC were, on average, tensile (expanding) in the anterior-posterior axis and compressive circumferentially. We found similar results when IOP was lowered by glaucoma eye drop administration in images taken 1 week apart.^31^ In addition, when eyes that had strain measures taken immediately after IOP-lowering were re-imaged 1 to 4 years later at the same range of lowered IOP, much greater strains were found, indicating the likelihood of remodeling effects over time.^32^

The present study performed strain measurements in living persons with induced increases in IOP to compare to our previous studies in which IOP had been lowered. To elevate IOP for 20 minutes, subjects wore modified swim goggles with lenses removed to allow both IOP measurement and imaging to expand upon a pilot study that demonstrated feasibility of the method.^17^ We included both healthy control subjects and patients with glaucoma with minimal to mild damage and with known findings in OCT retinal nerve fiber layer thickness and visual field testing. This much larger study permitted the comparison of eyes that had been stable in testing outcome with those who had significant past progressive worsening. The overall aim of this research was to determine if LC strain measurements represent a practical biomarker for susceptibility to glaucoma damage.

## Methods

### Recruitment of Subjects

The research was approved and supervised by the Institutional Review Board of the Johns Hopkins School of Medicine and abided by the tenets of the Declaration of Helsinki and can be found at ClinicalTrials.gov Identifier: NCT03267849. Written informed consent was obtained for each subject. Control persons were recruited as family members of subjects with glaucoma presenting to the Wilmer Glaucoma Center of Excellence, but were not biologically related to the glaucoma test subjects. Controls were examined at the slit lamp, had normal IOP (<18 millimeters of mercury [mm Hg]) and had normal ophthalmoscopic examinations with no evidence for glaucoma, nor any history of past treatments for glaucoma. One of the five control subjects was bilaterally pseudophakic, and the other four were phakic in tested eyes. Control eyes did not undergo OCT or visual field testing. Subjects with glaucoma were tested as part of a regularly scheduled visit to the Glaucoma Center. Subjects with glaucoma were either ocular hypertensive persons (history of IOP > 21) or those with glaucomatous optic nerve damage on OCT and visual field tests, as defined in Iyer et al.^33^

### Description of Goggles

The goggles used are produced by Speedo International (Lindon, UT, USA). They have individual eye covering areas with flexible material that pressed against the orbital tissues in each eye when placed with sufficient force. We removed the central lens material from the pairs of goggles that were selected to fit persons of different head sizes. The absence of the goggle lenses permitted both IOP measurement and OCT imaging with the goggles in place. The concept that wearing such goggles might represent a method to study temporary IOP elevation was published previously.^34^

### Description of Instruments Used

IOP was measured by the technical staff or physician on the day of testing by applanation tonometry, and then by the investigators using the iCare tonometer (iCare Finland Oy, Espoo, Finland) both prior to the application of goggles, immediately after application of the goggles, and (in many subjects) after wearing the goggles for 20 minutes with the goggles in place. For each measurement, the mean of six IOP determinations were averaged.

Imaging was performed with the Heidelberg Spectralis (Heidelberg Engineering GmbH, Heidelberg, Germany). Keratometry readings and axial length were measured using the IOL Master instrument (Zeiss, Dublin, CA, USA) and keratometry measurements were entered prior to each imaging set. First, images were collected without the wearing of goggles. Then, the goggles were placed and the IOP was measured to assure that it had been increased from baseline. Twenty minutes later, the IOP and keratometry were remeasured and the second set of images were taken with the goggles on.

### Description of Image Processing and Analysis

**Figure 1.**
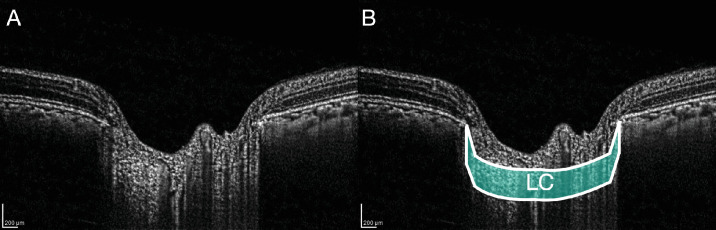
(**A**) Enhanced OCT image of the optic nerve head region in a B scan analyzed with contrast limited adaptive histogram equalization and gamma correction. (**B**) The segmented LC zone in color.

For each session, 24 radial OCT scans were acquired in enhanced depth imaging to improve depth resolution, each 768 × 495 pixels in (R, Z), centered on the ONH, with 7.5 degrees between adjacent scans, resulting in an approximate circumferential (Q) resolution of 108 µm/pixel at the lateral borders of the LC (Fig. 1). Anterior-posterior (Z) resolution was uniformly 3.87 µm/pixel, whereas radial (R) resolution varied from 5.33 to 6.94 µm/pixel. Up to three sequential image scans were taken at each session, with the pair having best contrast chosen for analysis. As in our previous publication, we compared two sequential sets of images from the baseline session to estimate the DVC baseline errors.^29^ Contrast of the OCT scans was enhanced by contrast-limited, adaptive piecewise histogram equalization (CLAHE) in FIJI (https:imageJ.net). To reduce noise, a gamma correction of 1.75 was applied to the image histogram. Settings were selected to minimize baseline strain error. Enhanced images were imported into MATLAB 2019a (Mathworks, Natick, MA, USA), reconstructed into a matrix of 8-bit intensity values for DVC.

### Image Segmentation

Masked observers (authors M.S.K. or R.M.) manually marked the two ends of Bruch's membrane on each side of the ONH and the anterior border of the LC at the baseline IOP using FIJI (see Fig. 1). Manually marked LC border positions were fit by piecewise linear interpolation to generate a smooth LC boundary. The intra-observer and inter-observer reproducibility of these marking methods was published.^35^ For the measurement of strains, the lateral borders of the LC were defined by two vertical lines projected downward from the ends of Bruch's membrane. The posterior border of the LC was defined by a curvilinear line parallel to the marked anterior LC border and 250 µm posterior to it (see Fig. 1). Because most often there is no visible contrast change at the myelin line, this selected LC thickness is based on histological measurements of the human LC.^36^

The LC region (see Fig. 1) was divided into the central and peripheral zones with the central diameter half of the Bruch's membrane opening diameter and also divided into four quadrants to analyze for relationships to retinal nerve fiber layer (RNFL) thickness.

### DVC Method to Estimate LC Strains and LC Depth Change

We modified the Fast Iterative DVC (FI-DVC) algorithm developed by Bar-Kochba et al.^37^ to analyze the radial OCT scans and calculate the displacement and strain response of the tissues. The theoretical foundation, implementation, and validation of the FI-DVC method were described in detail by Bar-Kochba et al.^37^ As for conventional DVC methods, the reference and deformed images are first divided into a grid of overlapping subsets. The FI-DVC method linearizes the deformation field mapping the reference to deformed images into *k* increments, such that at iteration k, the displacement field is the sum of all displacement increments of the prior iterations, that is, ***u****^k^***(*x*)** = ***u****^k−1^***(*x*)*** +* ***du****^k^***(*x*)**. For each iteration, the cross-correlation formulation is used to calculate ***du****^k^* for the subsets, and a window weighting function is applied to the cross-correlation formula to preserve the high spatial frequency information. The updated displacement field is used to warp the reference and deform image volumes closer together. In subsequent iterations, the subset size is decreased by a factor of two, and the weighted cross-correlation formulation is applied to the warped volumes to calculate the next displacement increment. The application of the window weighting function, iterative image deformation method, and iterative subset size refinement allow FI-DVC to capture large deformation and localized large strains more accurately than the conventional DVC method that uses a fixed subset size.

We modified the FI-DVC algorithm to analyze radial scans and investigated the accuracy and uncertainty of the calculated baseline error for the zero-strain condition and the correlation error for a superposed translation and uniform strain condition (Supplementary Information, Supplementary Tables S2A, S2B).^17^^,^^29^ The modified FI-DVC algorithm calculates the displacement components in the anterior-posterior direction (U_z_), radial direction (U_r_), and circumferential direction (U_q_). Displacements were filtered using a weighted correlation coefficient threshold of 0.055, defined in Bar-Kochba et al.^37^ where correlations matching below the threshold are excluded. Regions where the correlation coefficient was below the threshold corresponded to dark areas with low contrast. We also applied a displacement error filter that removed regions where the displacement correlation errors exceeded 5 µm, displacements on the edges of the radial scans, and displacement outliers as described in Midgett et al.^17^ The average percent area correlation of the LC region with accurate DVC correlation was 50.03 ± 20.28% area. In a prior sensitivity study, we showed that the threshold value 0.055 provided low baseline displacement and strain errors while maximizing the percent area of the LC region with accurate DVC correlation.

**Figure 2.**
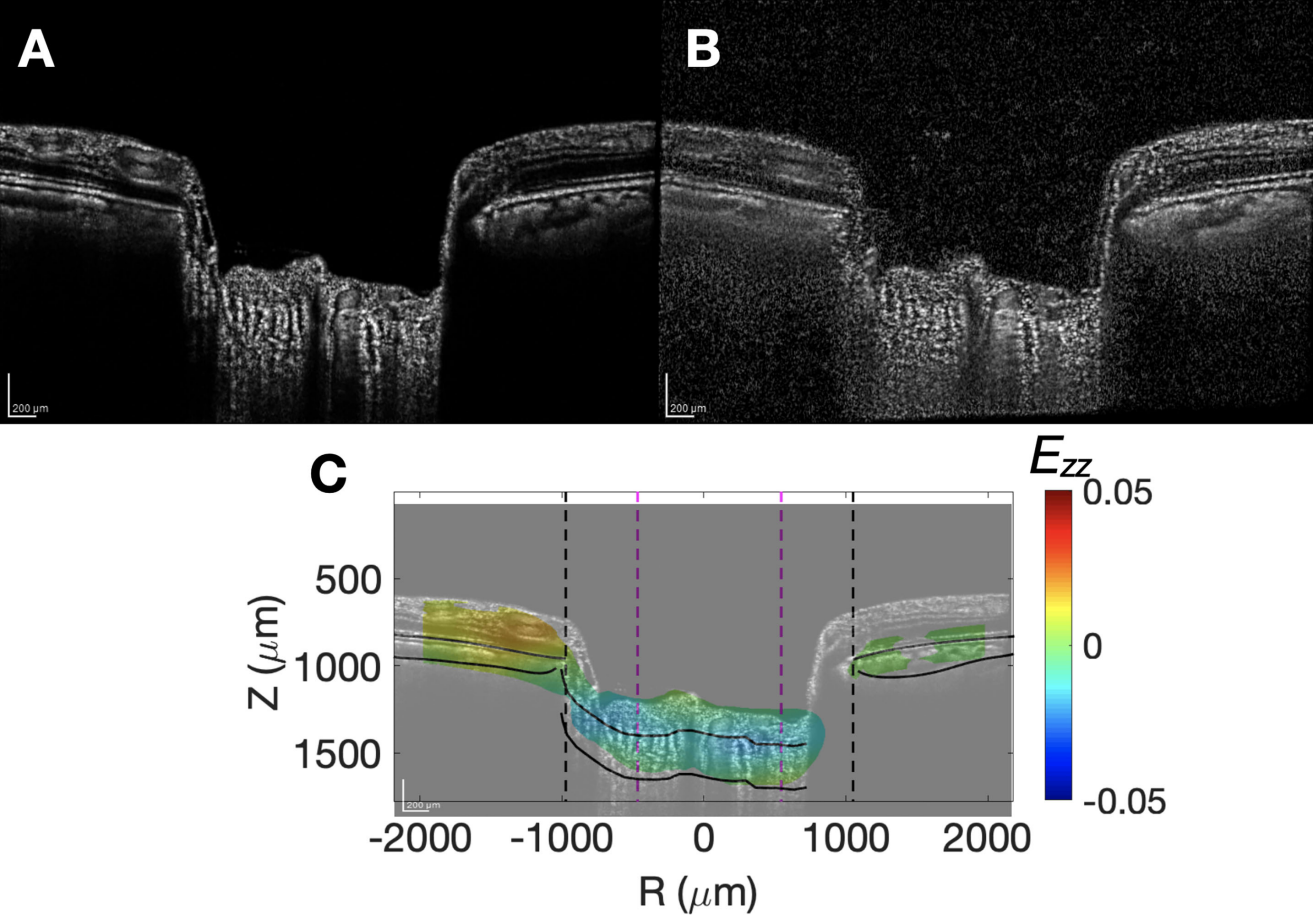
Images taken (**A**) before and (**B**) after goggles wearing. (**C**) The anterior-posterior strain map (*E_zz_*) indicating axial contraction (*blue*) in the lamina cribrosa and prelaminar areas.

The displacement field is smoothed, as described in Midgett et al.,^17^ to calculate the displacement gradient and components of the Green-Lagrange strain tensor in the cylindrical coordinate system (Fig. 2). Specifically, we calculated the strain components, *E_rr_*, *E_θθ_*, *E_zz_*, *E_r__θ_*, *E_z__θ_*, and *E_rz_*, in the cylindrical coordinate system, as well as the maximum principal strain (*E_max_*) and maximum shear strain *Γ_max_* in the RZ plane (OCT scan plane). Negative strains represent contractions, whereas positive strains represent expansions. The subscripts (*R*, *Z*, and *Θ*) represent the direction of strain. For example, a negative *E_zz_* strain in the LC represents local thinning in the anterior-posterior position. A positive *E_rr_* and *E_θθ_* strain represent expansion in the radial direction and circumferential directions, respectively. As an illustration, the combination (–*E_zz_*, +*E_rr_*, and +*E_θθ_*) would represent a 3-dimensional change in which a structure shaped like a soup can would deform into a tuna fish can. Shear strains (*E_r__θ_*, *E_z__θ_*, and *E_rz_*) represent the strain in two directions and are visualized as a rectangle being deformed into a diamond shape. Additionally, we measured the LC depth change across the entire visible LC border by measuring the movement of the LC border relative to the BMO from before to after wearing the goggles.^17^

The baseline strain error was calculated for each eye by performing DVC on two sets of back-to-back before images and represents the reproducibility error. The average baseline error for *E_zz_* were 0.021 ± 0.13%, which was in-line with previous studies of IOP-lowering.

### OCT and Visual Field Outcomes

OCT RNFL imaging was used to define structural loss in glaucoma subjects, with at least a 7 of 10 quality rating (Zeiss Cirrus OCT; Zeiss Meditec, Dublin, CA, USA). Visual fields were performed on Zeiss 24-2 Sita Standard field tests (Zeiss HFA 2i; Zeiss Meditec, Dublin, CA, USA). Past progressive change in OCT or field tests from the years prior to imaging was assessed by the Structure/Function GPA analysis of Zeiss Forum software. To qualify as prior change, the OCT or field progression designation of “possible” or “likely” had to be present on at least two tests without reverting to no progression at the most recent pair of tests.

### Outcomes to be Presented

We present averaged anterior LC depth (ALD) change and the strains, *E_zz_*, *E_θθ_*, *E_rr_*, *E_r__θ_*, *E_θ__z_*, *E_rz_*, and *E_max_* and *Γ_max_* in the R-Z plane from DVC analysis. DVC used OCT images taken before goggles as the reference state. As in previous work, ALD was defined as the displacement of the LC surface relative to the displacement of a line connecting the two points marking Bruch's membrane opening.^17^ Positive ALD was a more posterior displacement of the LC surface relative to Bruch's membrane. We divided each strain measure by the IOP increase for that eye to generate a parameter called compliance. An LC with high compliance would indicate that it would on average experience larger strain for a given IOP increase (i.e. a softer response) than one with low compliance. Data from all 57 goggles tests were used in a summary analysis, then data for eyes from persons with diagnosed glaucoma were calculated separately, along with the effects on strain in glaucoma eyes associated with RNFL and visual field status, as well as field progressive change in the years prior to testing. We also included assessment of the effects of age and axial length on ALD change, strains, and their compliance.

### Statistical Methods

The average values for primary outcomes were calculated. Due to the inclusion of both eyes from some subjects, the analysis was performed using generalized estimated equation regression models.^38^ The primary outcomes were compared for the entire LC, for the central and peripheral LC, and data divided into four quadrants (superior, inferior, nasal, and temporal). Linear regression was used to examine the relationships between IOP change and the average strains and the LC depth change. The relationship between outcomes and structural and functional measures of glaucoma damage was examined by comparing the RNFL thickness and visual field status of each eye. The value for statistical significance was taken as ≤ 0.05.

## Results

### Patient Population

Goggles tests and their imaging data analysis were successfully carried out in 57 eyes of 39 patients. These comprised 9 eyes of 5 healthy control persons with no evidence of glaucoma and 48 eyes of 33 persons who were either glaucoma suspects or patients with glaucoma. We consented other persons who did not complete the study or did not provide usable data. Among these 71 incomplete eyes, the failure to obtain data resulted from: (1) IOP did not increase at least 3 mm Hg on wearing the goggles; (2) the goggles could not be comfortably worn for 20 minutes; or (3) satisfactory images were not obtained at either baseline or after goggles wear.

For the 57 eyes that provided data, the average age of control persons was 64.2 ± 10.0 years (range = 47–71 years) and of the glaucoma group, the average age was 60.8 ± 13.0 years (range = 35–80 years, no difference between control and glaucoma group ages, *P* = 0.69). The control group had 2 men and 3 women, whereas the glaucoma group had 17 men and 16 women. The derivation of controls was three Europeans, one Asian, and one African American. For subjects with glaucoma, derivation was 16 Europeans, 12 African American, 3 Asians, 1 Hispanic, and 1 Other. There were no calculated differences in results by sex or derivation. In subjects with glaucoma, there were 7 pseudophakic eyes and 41 phakic eyes. Among subjects with glaucoma, 28 of 48 eyes were classified as glaucoma suspects due to ocular hypertension, whereas the other 20 were considered to have glaucomatous optic neuropathy, satisfying the criteria of a statistically abnormal superior or inferior quadrant in RNFL OCT with matching abnormality in 3 or more points in the opposite hemifield of the field test.^33^ Among all subjects, the mean axial length was 24.6 ± 1.6 mm, with only 7 eyes > 26 mm in length. The glaucoma group eyes consisted of 20 of 48 that were not on topical IOP lowering medication at the time of testing, whereas the other 28 were prescribed glaucoma eye drops. Only three eyes of two subjects with glaucoma had undergone past glaucoma surgery (bilateral trabeculectomy in 1 person and 1 tube shunt in another).

Visual field and OCT RNFL imaging were only performed on the glaucoma group eyes. At the most recent testing within 1 year of imaging, glaucoma eyes had mean mean deviation (MD) of –2.17 ± 3.18 decibels (dB), with a range of +1.42 to –11.97 dB (data from 46/48 eyes). Forty of the glaucoma group eyes had MD > –6 dB and none was worse than –12 dB. The mean Pattern Standard Deviation (PSD) was 3.37 ± 3.08 dB, and mean Visual Function Index (VFI) was 93.8 ± 9.03. The Glaucoma Hemifield Test result was outside the normal limits in 19 eyes, normal in 23 eyes, and graded as another outcome in 6 eyes. At the most recent OCT RNFL test, the mean RNFL average value was 80.4 ± 15.7 micrometers, with 15 of 48 eyes exceeding the statistical normality limit for mean RNFL (data from 48/48 eyes). In RNFL quadrant data, 16 eyes were statistically abnormal in the superior quadrant and 18 in the inferior quadrant.

Prior OCT and field testing among subjects with glaucoma were available for extended periods prior to the study imaging. This included a median of six past RNFL measurements (range = 1–10) and seven field tests (range = 1–21). Progressive worsening on RNFL and visual field were judged by the Glaucoma Progression Analysis software (Zeiss Forum; Zeiss Meditec, Dublin, CA, USA). In OCT data, glaucoma subject eyes were likely progressive in 9, possibly progressive in 18, and not progressive in 10 eyes (11 eyes had insufficient tests). Prior visual field tests identified 6 eyes with likely progression, 2 with possible progression, 28 with no progression, and 12 eyes with insufficient data. There was matching possible/definite RNFL progression in five of the six progressing field eyes.

IOP data are presented here for those eyes that provided strain data. For glaucoma eyes, the pre-imaging applanation IOP was 16.3 ± 4.6 mm Hg, the mean iCare IOP pre-imaging was 14.8 ± 5.1 mm Hg, and the iCare minus applanation mean difference was –1.5 ± 2.5 (*P* < 0.0001). The average IOP increase in glaucoma eyes from iCare tonometry pre-goggles to immediately after application of goggles was 7.1 ± 3.9 mm Hg (range = 3–16 mm Hg) and for controls it was 7.1 ± 3.6 mm Hg. In 32 eyes (containing both control and glaucoma eyes), the iCare IOP at the end of the 20 minutes of goggle wear was lower than at their application, with a mean difference of 2.9 ± 3.8 mm Hg.

## Results – Strain Outcomes

### Overall Subject Data

**Figure 3.**
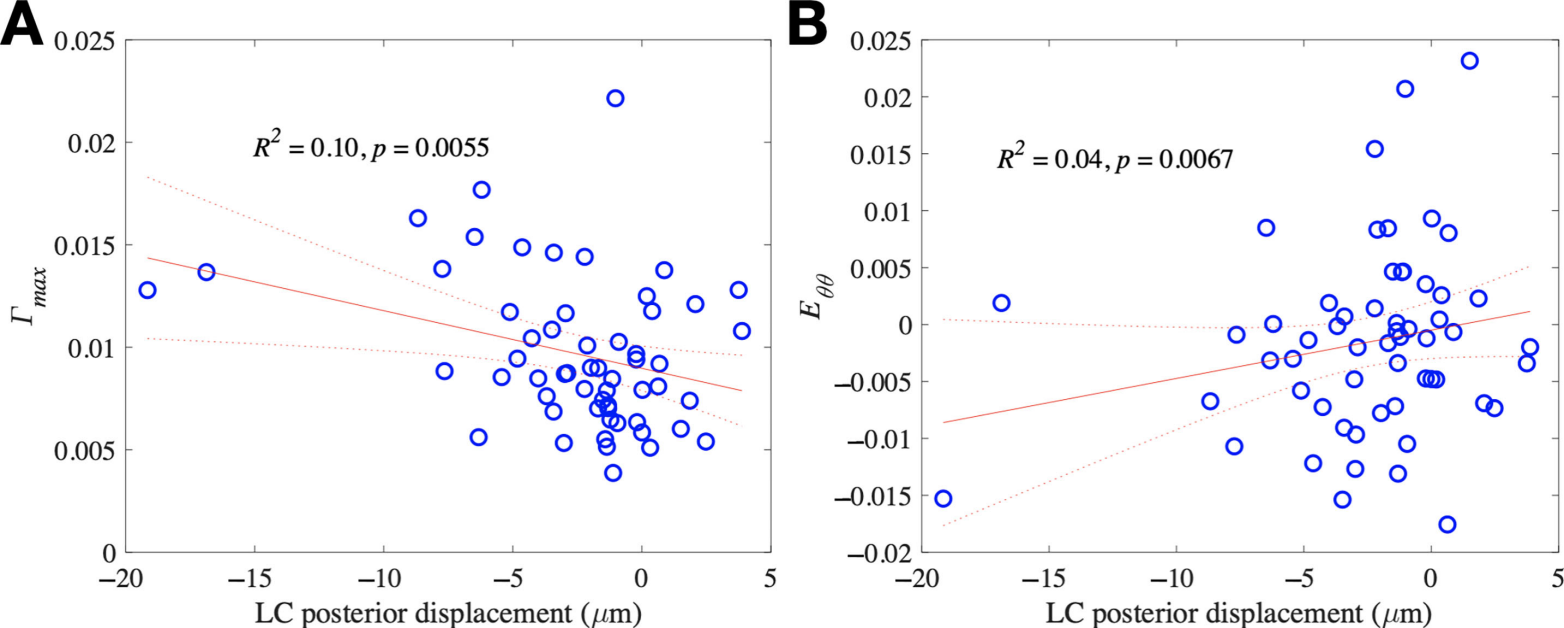
The associations between strains (*Γ_max_* and *E_θθ_*) and LC depth change (ALD). The more the ALD moved anteriorly (into the eye, negative posterior displacement), the greater the *Γ_max_* (*left*) and the more contractile the *E_θθ_* (*right*). Regression analysis significant with only modest explanation of overall relationships.

Data for all 57 eyes using generalized estimating equation (GEE) models to account for inclusion of both eyes in some subjects showed that IOP increase led to a mean movement into the eye, –2.49 µm, *P* = 0.0002), *E_zz_* strain = –0.37% (contractile, *P* < 0.0001), and significant *E_max_* and *Γ_max_* strains, 0.80% and 0.97%, respectively (both *P* < 0.0001; see the Table). In the overall group, we included the control subjects, untreated glaucoma suspects, and treated patients with glaucoma to provide a spectrum of responses. Detailed data for overall subjects and subgroups, including their GEE models, are included in Supplementary Tables S1 to S24. The primary outcomes for the study were findings in subjects with glaucoma, but the overall data are included here for completeness. For the overall group, *E_zz_* and *E_θθ_* were significantly different than baseline error (see Supplementary Tables S1, S2). One shear strain, *E_r__θ_*, showed a significant relation to the degree of IOP increase (*P* = 0.0083), but other strains did not (see Supplementary Table S3). The more the ALD moved anteriorly, the greater was the change in *Γ_max_* strain, the more contractile was *E_θθ_* strain, and the more positive *E_r__θ_* shear strain (*P* = 0.0055, *P* = 0.0067, and *P* = 0.014, respectively; Fig. 3, Supplementary Table S4). The *E_max_* and *E_zz_* strains in the peripheral LC were significantly more positive (tensile) compared to the central LC (*P* = 0.0013 and *P* < 0.0001, respectively). Strains *E_zz_* and *E_max_* and ALD did not differ by quadrant for the overall group (*P* > 0.2). Average strains and ALD change were not significantly related to age in models that included IOP change (all *P* > 0.08). We verified these models by using models comparing age to compliance (strain/IOP increase and ALD change), there were no significant age associations (all *P* > 0.06).

**Table.** LC Strains and Depth Change With IOP Increase From Goggles

	Mean	Standard	*P* Value
*E_zz_*	–0.0037	0.0063	**<0.0001**
*E_rr_*	0.0003	0.0038	0.61
*E_θθ_*	0.0015	0.0081	0.15
*E_r__θ_*	–0.0014	0.0067	0.12
*E_z__θ_*	0.0001	0.0068	0.93
*E_rz_*	0.0004	0.0024	0.25
*E_max_*	0.0080	0.0045	**<0.0001**
*Γ_max_*	0.0097	0.0036	**<0.0001**
*ALD*	–2.49	4.06	**0.0002**

The *P* values in boldface represent statistical significance.

Baseline IOP was not related to the initial axial length (*P* = 0.66). However, several strain outcomes were related to axial length. In eyes with longer axial length, ALD change was more anterior, *E_θθ_* was more compressive, and *E_rz_* was greater (*P* = 0.026, *P* = 0.0032, and *P* = 0.0078; see Supplementary Table S5).

### Data for the Overall Glaucoma Group

Strain data for the 48 eyes of 33 persons comprising the glaucoma group were analyzed separately from non-glaucoma eyes and could be compared to structural and functional parameters available in that cohort. Strains that were significantly different from zero were: contractile *E_zz_* (average = _–_0.33%, *P* = 0.0005), contractile *E_θθ_* (average = –0.23%, *P* = 0.03), *E_max_* (average = 0.83%, *P* < 0.0001), *Γ_max_* (average = 0.95%, *P* < 0.0001), and ALD change (average = 2.39 µm, representing anterior movement, *P* = 0.0002; see Supplementary Table S6). When strains were modeled with IOP as an independent variable, greater positive *E_r__θ_* was associated with greater IOP increase, whereas greater negative *E_r__θ_* was associated with smaller IOP increase (*P* = 0.015). In addition, greater *Γ_max_* (*P* = 0.02) and *E_max_* (*P* = 0.056) were associated with greater IOP increase (see Supplementary Table S7).

**Figure 4.**
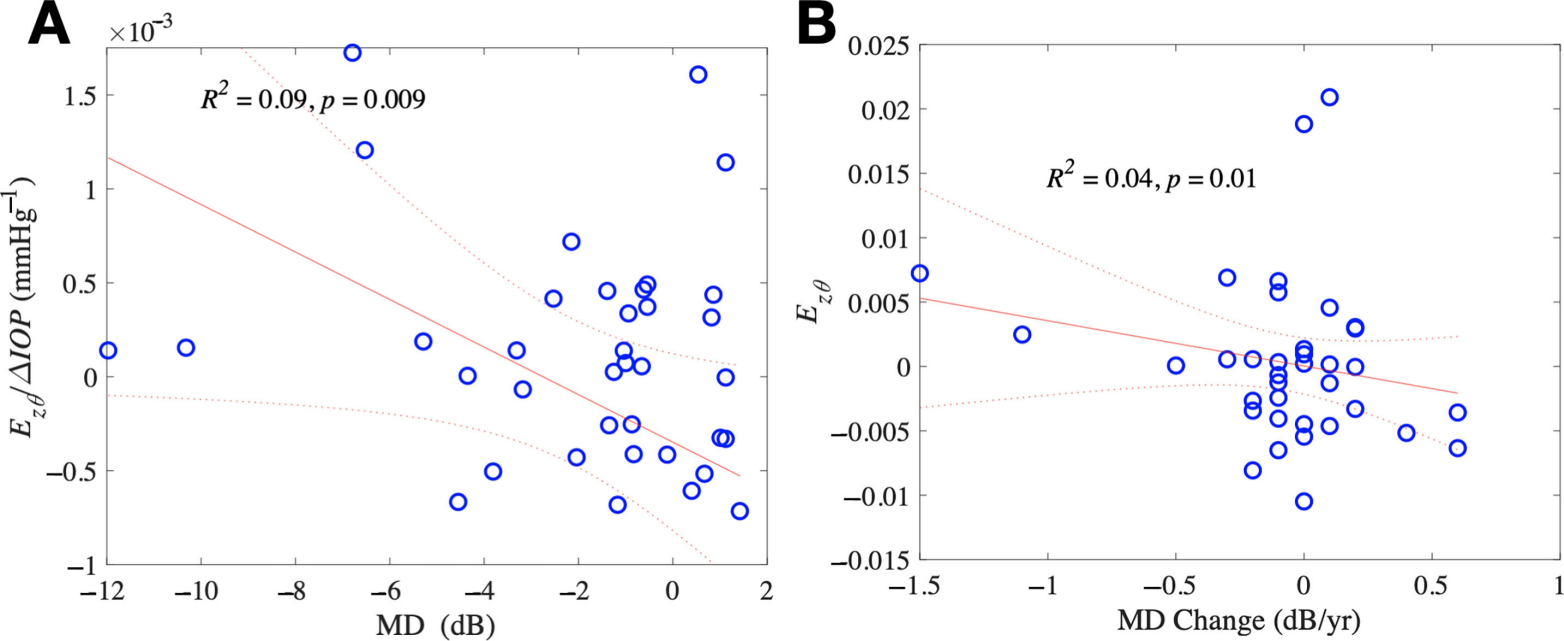
The associations between strain compliance and visual field at baseline and in previous testing. (**A**) Greater positive compliance of *E_z__θ_* was associated with more negative baseline MD (*left*). (**B**) Greater positive *E_z__θ_* was associated with greater MD decrease per year (*right*). Regression analysis significant with only modest explanation of overall relationships.

We defined strain compliance as the specimen strain divided by the increase in IOP, comparing strain compliance to the measures of glaucoma damage in the visual field and the OCT nerve fiber layer thickness. The compliance of *E_z__θ_* shear was highly associated with MD, where a more negative MD was associated with greater positive compliance of *E_z__θ_* (*P* = 0.009; Fig. 4, see Supplementary Table S8). Multivariate GEE models of MD and IOP increase showed the same trends for *E_z__θ_* (*P* = 0.044 vs. MD, *P* = 0.59 vs. IOP; see Supplementary Table S9). The same associations between strain compliance and VFI were found. A lower VFI (worse damage) was associated with greater positive compliance of *E_z__θ_* (*P* = 0.0009; see Supplementary Table S10). Multivariate GEE models of VFI and IOP increase confirmed these trends for *E_z__θ_* (*P* = 0.006 VFI, *P* = 0.60 IOP; see Supplementary Table S11). Multivariate GEE models of RNFL and increase IOP showed a smaller *Γ_max_* was associated with a thicker RNFL (*P* = 0.024 RNFL, *P* = 0.86 IOP; see Supplementary Table S12).

We compared strains and ALD change to the previous rate of change in field MD in the years prior to imaging. Only the *E_z__θ_* strain was significantly greater as MD rate of worsening increased (*P* = 0.01, *n* = 36; Fig. 4, see Supplementary Table S13). Strains were also modeled against rate of VFI worsening, with *E_z__θ_* and *E_zz_* each showing higher strain with progressively worsening VFI (*P* = 0.02 and *P* = 0.0005, *n* = 36; see Supplementary Table S14). Strain compliance was compared to the rate of MD worsening and several compliances were significantly related to MD rate, with *E_r__θ_*, *E_z__θ_*, and *E_θθ_* all increasing with greater worsening rate of MD (*P* = 0.04, *P* = 0.007, and *P* = 0.03, respectively). Further, *Γ_max_* compliance was less as MD rate was more negative (*P* = 0.03; see Supplementary Table S15). Only *E_z__θ_* compliance significantly increased with the rate of VFI worsening (*P* = 0.02; see Supplementary Table S16).

The strain compliance was compared between eyes that had previously undergone definite or possible progressive worsening in average OCT RNFL or in visual field (*n* = 30), compared to eyes with no progression (*n* = 10). The only significant difference was for *Γ_max_*, which was more compliant in eyes with RNFL progression or possible progression, compared to eyes with no progression (*P* = 0.0054). Compliance of strains was not different for eyes that used IOP-lowering eyedrops chronically and those that did not (*P* > 0.1).

### Data for Glaucoma Eyes With Definite Damage

Because the glaucoma group contained glaucoma suspects without detectable damage, as well as eyes with glaucomatous optic neuropathy, we conducted analyses for the 12 eyes with damage, defined as those with VFI indices < 95%. Among this group, VFI was not associated with compliance (*P* > 0.09; see Supplementary Table S17). However, multivariate models accounting for IOP and VFI showed higher VFI was associated with more contractile *E_rr_* (*P* = 0.005). In addition, the multivariable models indicated that lower VFI was associated with more positive *E_rz_* (*P* = 0.001; see Supplementary Table S18). MD was not associated with any strain compliance nor with ALD in the subgroup with VFI < 95%.

**Figure 5.**
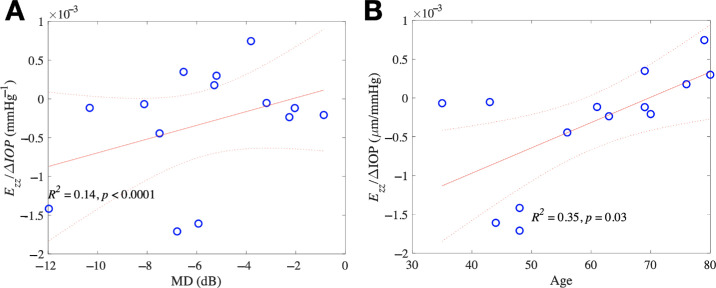
Models comparing strain compliance to MD in visual field, adjusted for age, among glaucoma eyes with abnormal mean RNFL thickness. More contractile compliance of *E_zz_* was associated with more negative MD (*left*), and younger age (*right*).

Finally, we constructed a second subgroup of damaged glaucoma eyes, namely the 14 eyes with mean RNFL thicknesses that were statistically abnormal compared to the normative database. In multivariate models with strain as the dependent variable and independent variables of RNFL and IOP change, thicker RNFL was associated with greater contractile *E_zz_* strain (*P* = 0.02) and thinner RNFL was associated with larger *E_max_* (*P* = 0.013) and *E_rr_* (*P* = 0.05; see Supplementary Table S19). Thicker RNFL was associated with greater contractile *E_zz_* compliance (*P* = 0.0001; see Supplementary Table S20). In these eyes with abnormal RNFL values, there was a more contractile *E_zz_* compliance for more negative MD (*P* < 0.0001; Fig. 5) and more positive *E_z__θ_* for more negative MD (*P* = 0.02; see Supplementary Table S21). Likewise, there was more contractile *E_zz_* compliance for worse VFI (*P* < 0.0001) and more positive *E_z__θ_* for worse VFI (*P* = 0.002; see Supplementary Table S22). When models including age were constructed, multivariate models of age, IOP change, and strain showed younger patients had greater contractile *E_zz_* (*P* = 0.01) and positive *E_θ__z_* (*P* = 0.007; see Supplementary Table S23). We verified this outcome using models comparing compliance (strain/ΔIOP) to age, indicating that younger age was associated with greater contractile *E_zz_* compliance (*P* = 0.03) and significantly positive *E_θ__z_* compliance (*P* < 0.0001; see Fig. 5, Supplementary Table S24).

## Discussion

In this report, we assessed the effect of IOP increase produced by wearing goggles for 20 minutes on LC strains. We determined the relationship of strains to the degree of IOP increase and to the degree of glaucoma damage and age. In all eyes studied (glaucoma and control), increased IOP was associated with axial contraction of the LC (negative *E_zz_*) relative to the baseline state and produced significant maximum principal strains, *E_max_* and *Γ_max_*. Further, the greater the IOP increase, the larger were *Γ_max_* and *E_max_* in GEE models in glaucoma eyes. This supports the validity of the strain estimates, which were greater than baseline errors. The maximum principal strain measured here was consistent with those reported by Fazio et al.^24^ for the ONH of brain-dead, younger non-glaucoma subjects. In that report, effective strain per mm Hg increase was 0.1%, which would correspond to approximately 0.7% for the average 7 mm Hg increase achieved by wearing the googles. We applied the equation for the effective strain in Girard et al.^25^ and calculated 0.923% effective strain for the goggles, which agrees with those of Fazio et al.^24^ Other studies applying ophthalmodynamometry to induce much larger IOP increases over very short time frames have found somewhat greater LC strains than those reported here.^26^^,^^27^ Specifically Beotra et al.^26^ reported an effective strain of 3.96% for a 30 mm Hg IOP elevation, which is approximately 4.3 times larger for 4 times the IOP increase.

Among glaucoma eyes, which represented the large majority of our subjects, there was significant axial contraction (*E_zz_*) with IOP increase. Higher IOP leading to axial LC compression (negative *E_zz_*) was expected, as there would be an increase in the translaminar pressure gradient. Indeed, in our two prior studies in which IOP was lowered, the axial expansion with IOP lowering was seen, as anticipated from the reduction in the translaminar pressure gradient, and greater level of tensile *E_zz_* was obtained for greater IOP lowering. However, a greater compressive *E_zz_* was not measured for higher IOP increase in this study. Change in the circumferential LC strain with goggles was a feature not identified with IOP lowering. Further, radial LC contraction was seen with IOP lowering, but not with goggles-induced IOP increase. With IOP-lowering by suture lysis, we found no significant differences in strains in the peripheral compared to the central LC, whereas IOP increase by goggle-wearing led to greater tensile strains peripherally than centrally. The failure to see ideally opposite findings with IOP change in different directions is possibly a result of the methods that we used to alter IOP. As opposed to suture lysis and eye drop administration, which only change IOP by altering aqueous outflow, the goggles produce their effect by compression of the orbital tissues around the eye. We speculate that this would both increase the translaminar pressure gradient (hence, causing LC axial contraction), as well as limiting expansion of the sclera. Thus, the goggles’ effects could lead to a reduction in the stress delivered to the LC by the peripapillary sclera—leading to the circumferential strain contraction not seen with IOP lowering. The effects of goggles on the sclera and the translaminar pressure gradient may complicate the relationship between IOP change and strains and explain the lack of statistical correlation. Studies that used an ophthalmodynamometer pressing against the sclera to induce an IOP increase also did not find a significant relationship between the strains and IOP change. The eye drop change approach is more likely to simulate the biomechanically relevant IOP effects in glaucomatous disease and we plan to test a larger group of eyes with change in eye drop use in a longitudinal, prospective study. IOP can be altered by either imaging before and after starting drops, or by a temporary washout of drop use in already treated persons.

When strains were modeled with IOP as the dependent variable in glaucoma eyes in this study, shear strains had quite significant associations with IOP change. For example, more positive *E_r__θ_* was associated with greater IOP increase, while greater negative *E_r__θ_* was associated with smaller IOP increase. We found that maximum shear strain (*Γ_max_*) was often greater than maximum principal strain (*E_max_*) in both overall and glaucoma groups. In our previous work, shear strains were nearly as large as normal strains and were associated with ALD change.^29^ As methods are further developed to estimate strains, care should be taken to investigate how shear strains could be important in producing short-term axonal damage and longer-term remodeling of the ONH.

To investigate whether strains were related to the degree of glaucoma injury, we calculated compliance of strains as strain/IOP increase and compared it to visual field and RNFL values. Again, the more substantial effects were seen with shear strains. Compliance of *E_z__θ_* shear strain was more positive in eyes with worse MD and VFI, confirming the findings after suture lysis and eye drop IOP lowering, for which greater compliances of several strains, including *E_max_*, *Γ_max_*, *E_zz_*, *E_r__θ_*, and *E_rz_*, were associated with worse visual field indices. After suture lysis, there were also greater compliances in eyes with thinner RNFL thickness for *E_max_*, *Γ_max_*, and *E_r__θ_*. When we examined the behavior of eyes with statistically abnormal RNFL thickness, their *E_zz_* and *E_z__θ_* compliances were greater as MD and VFI became more abnormal. Another group has detected greater strains in regions of glaucoma eyes with poorer retinal sensitivity and worse visual field loss.^27^

To examine further the relation of strain behavior to glaucoma damage, we compared the progression rate in MD and VFI during the years prior to imaging to the strain compliances. *E_r__θ_*, *E_z__θ_*, *E_θθ_*, and *Γ_max_* compliances increased with greater past worsening rate of MD, whereas *E_z__θ_* compliance was greater in eyes with greater VFI worsening. In addition, *Γ_max_* was more compliant in eyes with definite or possible progression in RNFL thickness. Finally, we examined GEE models for the 12 glaucoma eyes that had VFI indexes < 95%. Among these, lower (worse) VFI was associated with more positive *E_rz_*. This continues a series of observations from our IOP decrease or increase research that point to greater LC compliance and strains in eyes that have more glaucoma injury.

The change in ALD in these eyes was very small, as we also found in eyes after IOP-lowering. In each case, the movement was sometimes into the eye and sometimes outward, whether IOP was higher or lower. Some strain measures tracked with the change in ALD and many others did not. It has become very clear that the position of the anterior LC border may be seriously affected by the reference plane used to define it. Some investigators have suggested using alternatives to BMO, which may change as the choroid responds to IOP.^24^^,^^39^^,^^40^ Although the anterior LC border is easily seen in OCT images, its significance for mechanical effects on the LC axons and astrocytes would clearly be indirect, compared to actual measures of strain within the LC. Our method of estimating strains by comparing images before and after IOP change does not depend upon change in position of the BMO, because the DVC method automatically re-identifies the anterior LC border in post-IOP change images from its features in the initial images.

There is a recent published analysis of negative pressure goggles and their potential effects on IOP and ocular mechanics.^41^ This device is fundamentally different from the goggles used here and is reported to lower IOP. Our study did not seek to determine the safety of longer term wearing of goggles of the type we studied by patients with glaucoma.

Even more cogent are observations that we have made on the change in ALD over time since changing IOP. In our first paper on ALD change after suture lysis,^23^ images were generally taken days to weeks after IOP lowering. In that time frame, with IOP still low at second imaging, the ALD changes were 2 orders of magnitude larger than those in a study where the second image was made only 20 minutes after IOP lowering. In another report,^32^ for which second images were made 1 to 4 years after IOP lowering, the ALD change was also over 100 times larger than that immediately after either IOP lowering or by comparison to the values seen here, 20 minutes after IOP increase. In addition, IOP lowering led to uniform ALD movement into the eye with these long-term observations. It is now increasingly clear that early viscoelastic behavior is still ongoing during the minutes to hours after IOP change, and that the remodeling effects of the cellular and extracellular tissues of the ONH are much greater in the longer term. This leads to the conclusion that dynamic behavior of the LC tissues must be measured carefully over time after IOP alteration.

Whereas we found that the LC in younger patients was slightly more compliant than that of older patients in axial strain (*E_zz_*), strains and strain compliances were not significantly related to age in models that included IOP change. Because we studied older persons with glaucoma, the narrow range of ages may have been insufficient to detect differences across the whole age spectrum. We also found no differences in strains for those being prescribed daily glaucoma eye drops compared to untreated eyes. Because myopia is associated with glaucoma and may have different biomechanical behavior, we determined that eyes with longer axial length had greater anterior ALD movement, more compressive *E_θθ_*, and greater *E_rz_*.

Our research had some limitations. IOP measured at the end of the 20 minutes of goggle-wearing had decreased, as would be expected from a tonographic effect with constant external force. Thus, the actual exposure to increased IOP was less than that estimated from initial IOP on donning goggles. Despite this effect, consistent and statistically significant strains were measurable. The tonographic effect may explain the weaker associations between strains and IOP change here than in the suture lysis and glaucoma medication studies.^31^ Second, we calculated LC strains in the area from the anterior LC border to 250 µm posterior to it. This is necessary as many eyes have no discernable posterior LC border (i.e. at the myelin line). Histological measures of LC thickness support this estimate as including only LC tissue in most eyes.^36^ Third, the goggles wearing method was able to obtain strain data only in about half of consented subjects, due to either discomfort or poor image quality with the goggles in place. This would reduce the likelihood that the method would be a practical approach for standard office settings. By contrast, we show in another report that strains are easily measured 1 week after starting or stopping glaucoma IOP eye drops, an approach that could be widely adopted in clinical practice.^31^ Some areas of the ONH do not provide useful data due to the blocking effect of overlying retinal arteries and veins. Fourth, whereas the baseline IOP was similar among these patients, it is possible that if a higher baseline IOP were present, the nonlinear nature of strain compliance versus IOP change would be better estimated. Finally, we calculated the DVC baseline errors and correlation errors for an applied 0.5% strain level (see Supplementary Table S2B) and showed that *E_zz_* and *E_θθ_* were statistically greater than their respective baseline strain errors. Moreover, the strain components were greater in magnitude than their respective correlation strain errors for all but *E_zθ_*. However, the baseline and correlation strain errors were calculated for simple, homogeneous deformation states. The deformation of the ONH distorts the natural speckle pattern, and thus, the displacement and strain errors are likely higher, particularly in regions with large localized strains.

In conclusion, LC strains are measurable after modest, short-term IOP increases while wearing goggles. Axial LC compression occurred, as expected from IOP increase, and peripheral strain was greater than central strain. *Γ_max_* and *E_zθ_* shear strains and compliances were increased. Several strains were greater with worse existing glaucoma damage, as well as with greater visual field progressive worsening in the years prior to goggle testing.

## Supplementary Material

Supplement 1
