# Supplementary material for: The Strain Response to Intraocular Pressure Increase in the Lamina Cribrosa of Control Subjects and Glaucoma Patients
Source: Transl Vis Sci Technol. 2024 Dec 4;13(12):7. doi: 10.1167/tvst.13.12.7 (PMC11627119; doi:10.1167/tvst.13.12.7)
Supplement: Supplement 1 [file tvst-13-12-7_s001.docx]

**Supplemental Material**

The baseline error was calculated by applying DVC to two image volumes taken under nominally the same conditions. This resulting displacement field indicates the displacement bias (error) and uncertainty caused by the natural speckles and image quality characteristics, including the image contrast and noise. The DVC displacement error field was smoothed as described in the Methods section. The gradient of the smoothed displacement field was calculated to determine the baseline strain error.

Supplemental Table 1: Average baseline error and p-values comparing to average LC strain for all eyes.

|  | | |
| --- | --- | --- |
|  | Mean | *p*-value |
| *E_zz_* | 0.000213 | **0.01** |
| *E_rr_* | 0.0000463 | 0.07 |
| *E_θθ_* | 0.000224 | **0.04** |
| *E_rθ_* | -0.0000661 | 0.33 |
| *E_zθ_* | 0.0000242 | 0.22 |
| *E_rz_* | -0.000164 | 0.18 |

The correlation error was calculated by numerically warping the OCT volume of radial scans by a 2% tensile stretch in the *r* directions, a 2% compressive stretch in the *z* direction and a 10 μm displacement in the *Z* direction. DVC was applied to the warped and un-warped image to calculate the displacement field. The DVC displacement field was smoothed as described in the Methods section, and the gradient of the smoothed displacement field was calculated to determine strain. The correlation errors were calculated as the difference between the applied and DVC displacement and strain fields. The correlation error evaluates the effect of an anisotropic 2% strain distortion on the displacement and strain errors.

Supplemental Table 2A: Average DVC correlation error for a 2% applied strain and p-values comparing to average LC strain for all eyes.

|  | | |
| --- | --- | --- |
|  | Mean ± SD | *p*-value |
| *E_zz_* | 0.00493 ± 0.00152 | 0.12 |
| *E_rr_* | -0.000802 ± 0.000577 | 0.11 |
| *E_θθ_* | 0.00279 ± 0.000614 | 0.32 |
| *E_rθ_* | -0.0000135 ± 0.00030312 | 0.59 |
| *E_zθ_* | 0.000351 ± 0.00101974 | 0.49 |
| *E_rz_* | 0.0000289±0.000236 | 0.74 |

Supplemental Table 2B: Average DVC correlation error for a 0.5% applied strain, p-values comparing to average LC strain (middle column), and p-values comparing the absolute value of the LC strains to the absolute value of the correlation strain errors (right column) for 30 eyes. *The average *E_rz_* from goggle-wearing was smaller than the average correlation error.

|  | Mean ± SD | *p*-value comparing strains | *p*-value comparing strain magnitudes |
| --- | --- | --- | --- |
| *E_zz_* | 0.00122± 0.00045 | **<0.00001** | **0.0012** |
| *E_rr_* | -0.000318 ± 0.000288 | **0.0099** | **<0.00001** |
| *E_θθ_* | 0.000799 ± 0.000125 | 0.0924 | **<0.00001** |
| *E_rθ_* | 0.0000296 ± 0.000475, | 0.198 | **<0.00001** |
| *E_zθ_* | 0.000518 ± 0.000813 | 0.784 | **0.0001*** |
| *E_rz_* | 0.0000369 ± 0.000133 | 0.0871 | **<0.00001** |

To address the reviewer’s concern, we re-ran the correlation error estimate on 30 eyes, which included the same rigid body motion of a 10 mm displacement in the *Z* direction but with an applied 0.5% compressive *E_zz_* and 0.5% tensile *E_rr_* and *E_θθ_*. We have added this to the Supplemental Table 2B. “We also processed 30 of the eyes with a 10 mm displacement in the *Z* direction, a 0.5% tensile stretch in the radial and circumferential directions, and a 0.5% compressive stretch in the axial *Z* direction to estimate error for strains with a similar magnitude to the average LC strains measured for google-wearing. The correlation errors for the normal strains were *E_zz_* = 0.00122 ± 0.00045, *E_rr_* = -0.000318 ± 0.000288, and *E_θθ_* = 0.000799 ± 0.000125, which were 24%, 6%, and 16%, respectively, of the magnitude of the .5% applied strain. The 0.5% correlation strain errors were smaller than those for a 2% applied strain (p<0.0012). The E_zz_ and E_rr_ strains from goggle-wearing for the 30 subjects were statistically different than their corresponding 0.5% correlation strain errors (p<0.01). Since some of the strain components were negative, we also compared the magnitude (absolute value) of the strain components to their respective correlation errors and found that the strain magnitudes from goggle-wearing were statistically greater than the magnitude of the correlation errors for all components but Ez_θ_ (p< 0.012).

Supplemental Table 3: GEE of the effect of IOP increase on LC strain with effects for left and right eyes from the same patient for all eyes.

|  | | |
| --- | --- | --- |
|  | Estimate | *p*-value |
| *E_zz_* | 0.0001 | 0.82 |
| *E_rr_* | 0.0000 | 0.85 |
| *E_θθ_* | 0.0000 | 0.89 |
| *E_rθ_* | 0.0005 | **0.0083** |
| *E_zθ_* | 0.0003 | 0.25 |
| *E_rz_* | 0.0001 | 0.29 |
| *E_max_* | 0.0003 | 0.19 |
| *Γ_max_* | 0.0002 | 0.11 |
| *ALD* | -0.2606 | 0.15 |

Supplemental Table 4: GEE on the effect of ALD change on LC strains with effects for left and right eyes from the same patient for all eyes.

|  | | |
| --- | --- | --- |
|  | Estimate | *p*-value |
| *E_zz_* | >-0.00001 | 0.88 |
| *E_rr_* | 0.0001 | 0.30 |
| *E_θθ_* | 0.0005 | **0.0067** |
| *E_rθ_* | -0.0003 | **0.014** |
| *E_zθ_* | 0.0003 | 0.21 |
| *E_rz_* | <0.00001 | 0.69 |
| *E_max_* | -0.0002 | 0.08 |
| *Γ_max_* | -0.0002 | **0.0055** |

Supplemental Table 5: GEE on the effect of axial length on the LC strains (or ALD change) with effects for left and right eyes from the same patient for all eyes.

|  | | | |  | |  | |  |  | |  |
| --- | --- | --- | --- | --- | --- | --- | --- | --- | --- | --- | --- |
|  | Estimate | Standard error | Lower confidence Limits | | Upper confidence Limits | | Z | | | *p*-value | |
| *E_zz_* | 0.000614 | 0.000483 | -0.00033 | | 0.001561 | | 1.271764 | | | 0.20 | |
| *E_rr_* | -0.00039 | 0.00022 | -0.00082 | | 4.35E-05 | | -1.76212 | | | 0.078 | |
| *E_θθ_* | -0.00168 | 0.00057 | -0.0028 | | -0.00057 | | -2.95072 | | | **0.0032** | |
| *E_rθ_* | -0.00092 | 0.000842 | -0.00258 | | 0.000727 | | -1.09727 | | | 0.27 | |
| *E_zθ_* | -0.00054 | 0.000587 | -0.00169 | | 0.000611 | | -0.91874 | | | 0.36 | |
| *E_rz_* | 0.000466 | 0.000175 | 0.000122 | | 0.000809 | | 2.658624 | | | **0.0078** | |
| *E_max_* | 0.000195 | 0.000304 | -0.0004 | | 0.000792 | | 0.64162 | | | 0.52 | |
| *Γ_max_* | 0.000112 | 0.00032 | -0.00051 | | 0.000738 | | 0.350165 | | | 0.73 | |
| *ALD* | -1.32718 | 0.596922 | -2.49712 | | -0.15723 | | -2.22337 | | | **0.026** | |

Supplemental Table 6: LC strains and depth change with IOP increase from goggles for eyes with glaucoma.

|  | | |
| --- | --- | --- |
|  | Estimate | *p*-value |
| *E_zz_* | -0.0033 | **0.0005** |
| *E_rr_* | 0.00046 | 0.425 |
| *E_θθ_* | -0.0023 | **0.03** |
| *E_rθ_* | -0.0014 | 0.13 |
| *E_zθ_* | <0.0001 | 0.93 |
| *E_rz_* | 0.00045 | 0.20 |
| *E_max_* | 0.0083 | **<0.0001** |
| *Γ_max_* | 0.0095 | **<0.0001** |
| *ALD* | -2.39 | **0.0002** |

Supplemental Table 7: GEE of the effect of IOP increase on LC strain with effects for left and right eyes from the same patient for glaucoma eyes.

|  | | | |  | |  | |  |  | |  |
| --- | --- | --- | --- | --- | --- | --- | --- | --- | --- | --- | --- |
|  | Estimate | Standard error | Lower confidence Limits | | Upper confidence Limits | | Z | | | *p*-value | |
| *E_zz_* | 8.26E-06 | 0.000284 | -0.00055 | | 0.000566 | | 0.029054 | | | 0.98 | |
| *E_rr_* | 9.72E-05 | 0.000126 | -0.00015 | | 0.000345 | | 0.769667 | | | 0.44 | |
| *E_θθ_* | 0.000137 | 0.00027 | -0.00039 | | 0.000666 | | 0.506201 | | | 0.61 | |
| *E_rθ_* | 0.00052 | 0.000215 | 9.92E-05 | | 0.000942 | | 2.42152 | | | **0.015** | |
| *E_zθ_* | 0.000143 | 0.000259 | -0.00036 | | 0.000651 | | 0.552101 | | | 0.58 | |
| *E_rz_* | 3.73E-05 | 7.28E-05 | -0.00011 | | 0.00018 | | 0.512968 | | | 0.61 | |
| *E_max_* | 0.000385 | 0.000202 | -1E-05 | | 0.00078 | | 1.909353 | | | **0.056** | |
| *Γ_max_* | 0.000345 | 0.000153 | 4.57E-05 | | 0.000644 | | 2.25972 | | | **0.02** | |
| *ALD* | -0.15075 | 0.278583 | -0.69676 | | 0.395266 | | -0.54112 | | | 0.59 | |

Supplemental Table 8: GEE of the effect of mean deviation on LC compliance (strain/ΔIOP) (or ALD/ΔIOP) with effects for left and right eyes from the same patient for glaucoma eyes.

|  | | | |  | |  | |  |  | |  |
| --- | --- | --- | --- | --- | --- | --- | --- | --- | --- | --- | --- |
|  | Estimate | Standard error | Lower confidence Limits | | Upper confidence Limits | | Z | | | *p*-value | |
| *E_zz_/ΔIOP* | -5.5E-06 | 6.23E-05 | -0.00013 | | 0.000117 | | -0.0883 | | | 0.93 | |
| *E_rr_/ΔIOP* | 2.48E-05 | 3E-05 | -3.4E-05 | | 8.35E-05 | | 0.827275 | | | 0.41 | |
| *E_θθ_/ΔIOP* | -1.7E-05 | 3.5E-05 | -8.6E-05 | | 5.11E-05 | | -0.49915 | | | 0.62 | |
| *E_rθ_/ΔIOP* | -3.6E-05 | 3.9E-05 | -0.00011 | | 4.01E-05 | | -0.9323 | | | 0.35 | |
| *E_zθ_/ΔIOP* | -0.00013 | 4.86E-05 | -0.00022 | | -3.2E-05 | | -2.61391 | | | **0.009** | |
| *E_rz_/ΔIOP* | -1E-05 | 2.12E-05 | -5.2E-05 | | 3.11E-05 | | -0.49224 | | | 0.62 | |
| *E_max_/ΔIOP* | -2.6E-05 | 3.23E-05 | -8.9E-05 | | 3.73E-05 | | -0.80202 | | | 0.42 | |
| *Γ_max_/ΔIOP* | -3E-05 | 4.24E-05 | -0.00011 | | 5.32E-05 | | -0.70613 | | | 0.48 | |
| *ALD/ΔIOP* | 0.035138 | 0.026788 | -0.01737 | | 0.087642 | | 1.311692 | | | 0.19 | |

Supplemental Table 9: Multivariate GEE of the effects of IOP increase and mean deviation on LC strain with effects for left and right eyes from the same patient for glaucoma eyes.

DV = dependent variable, IV = Independent Variable, MD = mean deviation

| DV | Estimate | Standard error | Lower confidence Limits | Upper confidence Limits | Z | *p*-value | IV |
| --- | --- | --- | --- | --- | --- | --- | --- |
| *E_zz_* | 7.21E-05 | 0.000302 | -0.00052 | 0.000664 | 0.23899 | 0.81 | IOP |
| *E_zz_* | -0.00026 | 0.000259 | -0.00077 | 0.000244 | -1.01706 | 0.31 | MD |
| *E_rr_* | 8.94E-05 | 0.000129 | -0.00016 | 0.000343 | 0.691457 | 0.49 | IOP |
| *E_rr_* | 6.47E-05 | 0.000147 | -0.00022 | 0.000353 | 0.440342 | 0.66 | MD |
| *E_θθ_* | -1.7E-05 | 0.000215 | -0.00044 | 0.000405 | -0.0802 | 0.94 | IOP |
| *E_θθ_* | 6.26E-05 | 0.000245 | -0.00042 | 0.000543 | 0.255589 | 0.80 | MD |
| *E_rθ_* | 0.000425 | 0.000194 | 4.48E-05 | 0.000805 | 2.191082 | 0.028 | IOP |
| *E_rθ_* | -0.0001 | 0.00018 | -0.00045 | 0.00025 | -0.5666 | 0.57 | MD |
| *E_zθ_* | 0.000151 | 0.000283 | -0.0004 | 0.000706 | 0.534454 | 0.59 | IOP |
| *E_zθ_* | -0.00056 | 0.00028 | -0.00111 | -1.3E-05 | -2.00642 | **0.044** | MD |
| *E_rz_* | -5.1E-06 | 6.82E-05 | -0.00014 | 0.000129 | -0.07424 | 0.94 | IOP |
| *E_rz_* | -0.0001 | 9.6E-05 | -0.00029 | 8.43E-05 | -1.08199 | 0.28 | MD |
| *E_max_* | 0.000285 | 0.000186 | -8E-05 | 0.000649 | 1.532029 | 0.13 | IOP |
| *E_max_* | -0.00019 | 0.000142 | -0.00047 | 8.61E-05 | -1.35563 | 0.18 | MD |
| *Γ_max_* | 0.000274 | 0.000131 | 1.74E-05 | 0.000531 | 2.092798 | 0.036 | IOP |
| *Γ_max_* | -0.00018 | 0.00013 | -0.00043 | 7.85E-05 | -1.35434 | 0.18 | MD |
| *ALD* | -0.12521 | 0.279414 | -0.67285 | 0.422433 | -0.44811 | 0.65 | IOP |
| *ALD* | 0.160004 | 0.275556 | -0.38008 | 0.700084 | 0.580657 | 0.56 | MD |

Supplemental Table 10: GEE of the effect of VFI on LC compliance (strain/ΔIOP) (or ALD/ΔIOP) with effects for left and right eyes from the same patient for glaucoma eyes.

|  | Estimate | Standard error | Lower confidence Limits | Upper confidence Limits | Z | *p*-value |
| --- | --- | --- | --- | --- | --- | --- |
| *E_zz_/ΔIOP* | -3.7E-06 | 1.83E-05 | -4E-05 | 3.22E-05 | -0.20302 | 0.84 |
| *E_rr_/ΔIOP* | 2.51E-06 | 8.62E-06 | -1.4E-05 | 1.94E-05 | 0.290784 | 0.77 |
| *E_θθ_/ΔIOP* | -1.6E-05 | 1.23E-05 | -4E-05 | 7.96E-06 | -1.31446 | 0.19 |
| *E_rθ_/ΔIOP* | -1E-05 | 1.34E-05 | -3.6E-05 | 1.59E-05 | -0.77053 | 0.44 |
| *E_zθ_/ΔIOP* | -6.2E-05 | 1.86E-05 | -9.8E-05 | -2.5E-05 | -3.30934 | **0.0009** |
| *E_rz_/ΔIOP* | -5.5E-06 | 6.68E-06 | -1.9E-05 | 7.61E-06 | -0.82041 | 0.41 |
| *E_max_/ΔIOP* | -1.3E-05 | 1.08E-05 | -3.5E-05 | 8.03E-06 | -1.22023 | 0.22 |
| *Γ_max_/ΔIOP* | -1.2E-05 | 1.42E-05 | -4E-05 | 1.57E-05 | -0.8571 | 0.39 |
| *ALD/ΔIOP* | 0.01299 | 0.007987 | -0.00266 | 0.028644 | 1.626433 | 0.10 |

Supplemental Table 11: Multivariate GEE of the effects of IOP increase and VFI on LC strain with effects for left and right eyes from the same patient for glaucoma eyes.

DV = dependent variable, IV = Independent Variable, VFI = visual function index

| DV | Estimate | Standard error | Lower confidence Limits | Upper confidence Limits | Z | *p*-value | IV |
| --- | --- | --- | --- | --- | --- | --- | --- |
| *E_zz_* | 8.71E-05 | 0.000313 | -0.00053 | 0.0007 | 0.278631 | 0.78 | IOP |
| *E_zz_* | -8.5E-05 | 7E-05 | -0.00022 | 5.24E-05 | -1.21101 | 0.23 | VFI |
| *E_rr_* | 8.55E-05 | 0.000132 | -0.00017 | 0.000344 | 0.649727 | 0.52 | IOP |
| *E_rr_* | -7.3E-06 | 4.42E-05 | -9.4E-05 | 7.94E-05 | -0.16612 | 0.87 | VFI |
| *E_θθ_* | -2.3E-05 | 0.000222 | -0.00046 | 0.000412 | -0.10508 | 0.92 | IOP |
| *E_θθ_* | -3.5E-05 | 6.94E-05 | -0.00017 | 0.000101 | -0.49878 | 0.62 | VFI |
| *E_rθ_* | 0.000427 | 0.000194 | 4.56E-05 | 0.000808 | 2.194483 | 0.028 | IOP |
| *E_rθ_* | -2.3E-05 | 6.27E-05 | -0.00015 | 9.94E-05 | -0.37367 | 0.71 | VFI |
| *E_zθ_* | 0.000148 | 0.000284 | -0.00041 | 0.000704 | 0.521145 | 0.60 | IOP |
| *E_zθ_* | -0.00027 | 9.79E-05 | -0.00046 | -7.8E-05 | -2.75584 | **0.006** | VFI |
| *E_rz_* | -4.9E-06 | 6.69E-05 | -0.00014 | 0.000126 | -0.0738 | 0.94 | IOP |
| *E_rz_* | -4E-05 | 3.17E-05 | -0.0001 | 2.19E-05 | -1.26813 | 0.20 | VFI |
| *E_max_* | 0.000286 | 0.000192 | -9E-05 | 0.000662 | 1.49143 | 0.14 | IOP |
| *E_max_* | -6E-05 | 4.68E-05 | -0.00015 | 3.22E-05 | -1.2724 | 0.20 | VFI |
| *Γ_max_* | 0.000277 | 0.000134 | 1.3E-05 | 0.00054 | 2.056948 | 0.040 | IOP |
| *Γ_max_* | -5.3E-05 | 3.64E-05 | -0.00012 | 1.82E-05 | -1.45976 | 0.14 | VFI |
| *ALD* | -0.15007 | 0.277905 | -0.69475 | 0.394614 | -0.54 | 0.59 | IOP |
| *ALD* | 0.007498 | 0.071533 | -0.1327 | 0.147701 | 0.104823 | 0.92 | VFI |

Supplemental Table 12: Multivariate GEE of the effects of IOP increase and RNFL on LC strain with effects for left and right eyes from the same patient for glaucoma eyes.

DV = dependent variable, IV = Independent Variable, RNFL = retinal nerve fiber layer

| DV | Estimate | Standard error | Lower confidence Limits | Upper confidence Limits | Z | *p*-value | IV |
| --- | --- | --- | --- | --- | --- | --- | --- |
| *E_zz_* | -0.00014 | 6.09E-05 | -0.00026 | -1.7E-05 | -2.24114 | 0.025 | IOP |
| *E_zz_* | -3E-05 | 0.000249 | -0.00052 | 0.000459 | -0.12134 | 0.90 | RNFL |
| *E_rr_* | 3.39E-05 | 3.56E-05 | -3.6E-05 | 0.000104 | 0.952024 | 0.34 | IOP |
| *E_rr_* | 0.000101 | 0.000126 | -0.00015 | 0.000347 | 0.799965 | 0.42 | RNFL |
| *E_θθ_* | 9.19E-05 | 6.2E-05 | -3E-05 | 0.000213 | 1.480609 | 0.14 | IOP |
| *E_θθ_* | 0.000153 | 0.00026 | -0.00036 | 0.000662 | 0.587288 | 0.56 | RNFL |
| *E_rθ_* | 1.11E-05 | 5.74E-05 | -0.0001 | 0.000124 | 0.193403 | 0.85 | IOP |
| *E_rθ_* | 0.000521 | 0.000216 | 9.67E-05 | 0.000945 | 2.407081 | 0.016 | RNFL |
| *E_zθ_* | -3.5E-06 | 7.94E-05 | -0.00016 | 0.000152 | -0.04401 | 0.96 | IOP |
| *E_zθ_* | 0.000143 | 0.000261 | -0.00037 | 0.000654 | 0.548978 | 0.58 | RNFL |
| *E_rz_* | -1.6E-05 | 2.13E-05 | -5.8E-05 | 2.56E-05 | -0.76077 | 0.45 | IOP |
| *E_rz_* | 3.62E-05 | 7.26E-05 | -0.00011 | 0.000178 | 0.498348 | 0.62 | RNFL |
| *E_max_* | -4.6E-05 | 3.51E-05 | -0.00011 | 2.28E-05 | -1.30826 | 0.19 | IOP |
| *E_max_* | 0.000382 | 0.000198 | -6.1E-06 | 0.000771 | 1.929236 | 0.054 | RNFL |
| *Γ_max_* | -6.3E-06 | 3.68E-05 | -7.8E-05 | 6.58E-05 | -0.17086 | 0.86 | IOP |
| *Γ_max_* | 0.000342 | 0.000152 | 4.48E-05 | 0.00064 | 2.25479 | **0.024** | RNFL |
| *ALD* | 0.229018 | 0.130519 | -0.02679 | 0.48483 | 1.754679 | 0.079 | IOP |
| *ALD* | -0.08414 | 0.202407 | -0.48085 | 0.31257 | -0.4157 | 0.68 | RNFL |

Supplemental Table 13: GEE of the effect of MD decrease per year on LC strain with effects for left and right eyes from the same patient for glaucoma eyes.

|  | Estimate | Standard error | Lower confidence Limits | Upper confidence Limits | Z | *p*-value |
| --- | --- | --- | --- | --- | --- | --- |
| *E_zz_* | -0.00189 | 0.001384 | -0.0046 | 0.000823 | -1.36539 | 0.17 |
| *E_rr_* | -0.00124 | 0.001498 | -0.00418 | 0.001693 | -0.83011 | 0.41 |
| *E_θθ_* | -0.00113 | 0.002658 | -0.00634 | 0.004079 | -0.42559 | 0.67 |
| *E_rθ_* | -0.0017 | 0.001586 | -0.00481 | 0.001407 | -1.07298 | 0.28 |
| *E_zθ_* | -0.00349 | 0.001343 | -0.00612 | -0.00086 | -2.60138 | **0.01** |
| *E_rz_* | -0.00203 | 0.001138 | -0.00425 | 0.000204 | -1.78035 | 0.075 |
| *E_max_* | -0.00168 | 0.001473 | -0.00457 | 0.001202 | -1.14385 | 0.25 |
| *Γ_max_* | 0.0003 | 0.001501 | -0.00264 | 0.003242 | 0.200044 | 0.84 |
| *ALD* | 1.20131 | 1.143612 | -1.04013 | 3.442749 | 1.050452 | 0.29 |

Supplemental Table 14 GEE of the effect of VFI decrease per year on LC strain with effects for left and right eyes from the same patient for glaucoma eyes.

|  | Estimate | Standard error | Lower confidence Limits | Upper confidence Limits | Z | *p*-value |
| --- | --- | --- | --- | --- | --- | --- |
| *E_zz_* | -0.00086 | 0.000246 | -0.00134 | -0.00038 | -3.48862 | **0.0005** |
| *E_rr_* | -0.00015 | 0.000405 | -0.00094 | 0.000646 | -0.36409 | 0.72 |
| *E_θθ_* | -0.00046 | 0.000745 | -0.00192 | 0.001 | -0.61721 | 0.54 |
| *E_rθ_* | -2.8E-05 | 0.000591 | -0.00119 | 0.001131 | -0.0479 | 0.96 |
| *E_zθ_* | -0.00174 | 0.000754 | -0.00322 | -0.00026 | -2.31083 | **0.02** |
| *E_rz_* | -0.00022 | 0.000397 | -0.001 | 0.000555 | -0.56014 | 0.58 |
| *E_max_* | -0.0007 | 0.000414 | -0.00151 | 0.000114 | -1.6838 | 0.092 |
| *Γ_max_* | -0.00031 | 0.000505 | -0.0013 | 0.000678 | -0.61855 | 0.54 |
| *ALD* | 0.047172 | 0.303749 | -0.54817 | 0.64251 | 0.1553 | 0.88 |

Supplemental Table 15: GEE of the effect of MD decrease per year on LC compliance (or ALD compliance) with effects for left and right eyes from the same patient for glaucoma eyes.

|  | Estimate | Standard error | Lower confidence Limits | Upper confidence Limits | Z | *p*-value |
| --- | --- | --- | --- | --- | --- | --- |
| *E_zz_/ΔIOP* | -0.00035 | 0.000317 | -0.00098 | 0.000266 | -1.11904 | 0.26 |
| *E_rr_/ΔIOP* | -0.00029 | 0.000272 | -0.00083 | 0.000241 | -1.07469 | 0.28 |
| *E_θθ_/ΔIOP* | -0.00063 | 0.000295 | -0.00121 | -5.1E-05 | -2.13359 | **0.03** |
| *E_rθ_/ΔIOP* | -0.00046 | 0.000218 | -0.00089 | -3.2E-05 | -2.1055 | **0.04** |
| *E_zθ_/ΔIOP* | -0.00069 | 0.000258 | -0.0012 | -0.00019 | -2.68377 | **0.007** |
| *E_rz_/ΔIOP* | -0.00027 | 0.000239 | -0.00074 | 0.000199 | -1.12756 | 0.26 |
| *E_max_/ΔIOP* | 0.000161 | 0.000283 | -0.00039 | 0.000716 | 0.567016 | 0.57 |
| *Γ_max_/ΔIOP* | 0.000506 | 0.00023 | 5.44E-05 | 0.000958 | 2.195936 | **0.03** |
| *ALD/ΔIOP* | 0.030806 | 0.233727 | -0.42729 | 0.488903 | 0.131804 | 0.90 |

Supplemental Table 16: GEE of the effect of VFI decrease per year on LC compliance (or ALD compliance) with effects for left and right eyes from the same patient for glaucoma eyes.

|  | Estimate | Standard error | Lower confidence Limits | Upper confidence Limits | Z | *p*-value |
| --- | --- | --- | --- | --- | --- | --- |
| *E_zz_/ΔIOP* | -0.00014 | 8.72E-05 | -0.00031 | 2.91E-05 | -1.62609 | 0.10 |
| *E_rr_/ΔIOP* | 6.46E-06 | 4.95E-05 | -9.1E-05 | 0.000104 | 0.130399 | 0.90 |
| *E_θθ_/ΔIOP* | -0.00015 | 9.64E-05 | -0.00034 | 3.9E-05 | -1.55517 | 0.12 |
| *E_rθ_/ΔIOP* | -2.6E-05 | 9.16E-05 | -0.00021 | 0.000154 | -0.2809 | 0.78 |
| *E_zθ_/ΔIOP* | -0.00032 | 0.000137 | -0.00059 | -5E-05 | -2.32263 | **0.02** |
| *E_rz_/ΔIOP* | 1.96E-05 | 5.03E-05 | -7.9E-05 | 0.000118 | 0.389563 | 0.70 |
| *E_max_/ΔIOP* | -1.2E-05 | 8.07E-05 | -0.00017 | 0.000146 | -0.14535 | 0.88 |
| *Γ_max_/ΔIOP* | 7.59E-05 | 8.23E-05 | -8.5E-05 | 0.000237 | 0.922485 | 0.36 |
| *ALD/ΔIOP* | 0.094385 | 0.059029 | -0.02131 | 0.210079 | 1.598953 | 0.11 |

Supplemental Table 17: VFI < 95 subgroup: GEE of the effect of VFI on LC compliance (or ALD compliance) with effects for left and right eyes from the same patient for glaucoma eyes with VFI < 95.

|  | Estimate | Standard error | Lower confidence Limits | Upper confidence Limits | Z | *p*-value |
| --- | --- | --- | --- | --- | --- | --- |
| *E_zz_/ΔIOP* | -0.00017 | 0.000167 | -0.0005 | 0.000156 | -1.02388 | 0.31 |
| *E_rr_/ΔIOP* | -3.8E-05 | 9.67E-05 | -0.00023 | 0.000151 | -0.39342 | 0.69 |
| *E_θθ_/ΔIOP* | -8.1E-05 | 0.000106 | -0.00029 | 0.000126 | -0.76391 | 0.44 |
| *E_rθ_/ΔIOP* | 3.57E-06 | 0.00017 | -0.00033 | 0.000336 | 0.021005 | 0.98 |
| *E_zθ_/ΔIOP* | 0.000133 | 0.000109 | -8.1E-05 | 0.000347 | 1.221649 | 0.22 |
| *E_rz_/ΔIOP* | -7.3E-05 | 4.36E-05 | -0.00016 | 1.21E-05 | -1.68218 | 0.093 |
| *E_max_/ΔIOP* | 8.07E-06 | 7.11E-05 | -0.00013 | 0.000147 | 0.113576 | 0.91 |
| *Γ_max_/ΔIOP* | 0.00013 | 8.96E-05 | -4.6E-05 | 0.000305 | 1.448956 | 0.15 |
| *ALD/ΔIOP* | 0.024188 | 0.0626 | -0.09851 | 0.146883 | 0.386393 | 0.70 |

Supplemental Table 18: VFI < 95 subgroup: Multivariate GEE of the effects of IOP increase and VFI on LC strain with effects for left and right eyes from the same patient for glaucoma eyes with VFI < 95.

DV = dependent variable, IV = Independent Variable, VFI = visual function index

| DV | Estimate | Standard error | Lower confidence Limits | Upper confidence Limits | Z | *p*-value | IV |
| --- | --- | --- | --- | --- | --- | --- | --- |
| *E_zz_* | -3.2E-05 | 0.000357 | -0.00073 | 0.000668 | -0.08996 | 0.92 | IOP |
| *E_zz_* | -0.00142 | 0.000861 | -0.00311 | 0.000266 | -1.65066 | 0.099 | VFI |
| *E_rr_* | 0.000544 | 0.000113 | 0.000323 | 0.000765 | 4.82519 | <0.0001 | IOP |
| *E_rr_* | 0.000735 | 0.00026 | 0.000225 | 0.001245 | 2.826735 | **0.005** | VFI |
| *E_θθ_* | 0.000226 | 0.000335 | -0.00043 | 0.000882 | 0.675 | 0.50 | IOP |
| *E_θθ_* | 0.000215 | 0.000741 | -0.00124 | 0.001668 | 0.290456 | 0.77 | VFI |
| *E_rθ_* | 0.000502 | 0.000354 | -0.00019 | 0.001196 | 1.418495 | 0.16 | IOP |
| *E_rθ_* | 0.00047 | 0.000857 | -0.00121 | 0.002149 | 0.54836 | 0.58 | VFI |
| *E_zθ_* | 0.000474 | 0.000363 | -0.00024 | 0.001184 | 1.306882 | 0.19 | IOP |
| *E_zθ_* | 0.001348 | 0.000761 | -0.00014 | 0.00284 | 1.77189 | 0.076 | VFI |
| *E_rz_* | -0.00016 | 8.96E-05 | -0.00033 | 1.91E-05 | -1.74691 | 0.081 | IOP |
| *E_rz_* | -0.00063 | 0.00019 | -0.001 | -0.00025 | -3.29564 | **0.001** | VFI |
| *E_max_* | 0.000231 | 0.000245 | -0.00025 | 0.000711 | 0.941706 | 0.35 | IOP |
| *E_max_* | -0.0005 | 0.000516 | -0.00151 | 0.000514 | -0.96414 | 0.33 | VFI |
| *Γ_max_* | 0.000244 | 0.000162 | -7.4E-05 | 0.000562 | 1.503719 | 0.13 | IOP |
| *Γ_max_* | 2.02E-05 | 0.000417 | -0.0008 | 0.000838 | 0.048384 | 0.96 | VFI |
| *ALD* | -0.23251 | 0.129478 | -0.48628 | 0.021264 | -1.79574 | 0.073 | IOP |
| *ALD* | 0.469721 | 0.376012 | -0.26725 | 1.206691 | 1.249215 | 0.21 | VFI |

Supplemental Table 19: RED RNFL subgroup: Multivariate GEE of the effects of IOP increase and RNFL on LC strain with effects for left and right eyes from the same patient for glaucoma eyes with red RNFL measurements.

DV = dependent variable, IV = Independent Variable, RNFL = retinal nerve fiber layer

| DV | Estimate | Standard error | Lower confidence Limits | Upper confidence Limits | Z | *p*-value | IV |
| --- | --- | --- | --- | --- | --- | --- | --- |
| *E_zz_* | 0.000375 | 0.000267 | -0.00015 | 0.000898 | 1.406685 | 0.16 | IOP |
| *E_zz_* | -0.00035 | 0.000148 | -0.00064 | -5.4E-05 | -2.32429 | **0.02** | RNFL |
| *E_rr_* | -6.2E-05 | 0.000104 | -0.00027 | 0.000142 | -0.5976 | 0.55 | IOP |
| *E_rr_* | -0.00018 | 9.08E-05 | -0.00035 | 1.17E-06 | -1.94713 | **0.05** | RNFL |
| *E_θθ_* | -0.00029 | 0.000221 | -0.00072 | 0.000144 | -1.31001 | 0.19 | IOP |
| *E_θθ_* | 6.98E-05 | 0.000173 | -0.00027 | 0.000409 | 0.402571 | 0.69 | RNFL |
| *E_rθ_* | 0.000292 | 9.84E-05 | 9.9E-05 | 0.000485 | 2.965531 | 0.0030 | IOP |
| *E_rθ_* | 0.000129 | 0.000273 | -0.00041 | 0.000663 | 0.472874 | 0.64 | RNFL |
| *E_zθ_* | -0.00045 | 0.000306 | -0.00105 | 0.00015 | -1.47108 | 0.14 | IOP |
| *E_zθ_* | -9.2E-05 | 0.000607 | -0.00128 | 0.001097 | -0.15198 | 0.88 | RNFL |
| *E_rz_* | 1.17E-05 | 0.000122 | -0.00023 | 0.00025 | 0.096006 | 0.92 | IOP |
| *E_rz_* | 1.03E-05 | 8.02E-05 | -0.00015 | 0.000168 | 0.128053 | 0.90 | RNFL |
| *E_max_* | 0.00039 | 0.000301 | -0.0002 | 0.000981 | 1.294955 | 0.20 | IOP |
| *E_max_* | -0.00038 | 0.000152 | -0.00068 | -8E-05 | -2.4885 | **0.013** | RNFL |
| *Γ_max_* | 0.000217 | 0.000203 | -0.00018 | 0.000615 | 1.067775 | 0.29 | IOP |
| *Γ_max_* | -0.00011 | 0.00012 | -0.00034 | 0.000127 | -0.90294 | 0.37 | RNFL |
| *ALD* | -0.64899 | 0.219357 | -1.07892 | -0.21905 | -2.95859 | 0.0031 | IOP |
| *ALD* | -0.00791 | 0.138916 | -0.28018 | 0.264359 | -0.05696 | 0.95 | RNFL |

Supplemental Table 20: RED RNFL subgroup: GEE of the effects of RNFL on LC compliance (or ALD compliance) with effects for left and right eyes from the same patient for glaucoma eyes with red RNFL measurements.

|  | Estimate | Standard error | Lower confidence Limits | Upper confidence Limits | Z | *p*-value |
| --- | --- | --- | --- | --- | --- | --- |
| *E_zz_/ΔIOP* | -5.1E-05 | 1.34E-05 | -7.8E-05 | -2.5E-05 | -3.8363 | **0.0001** |
| *E_rr_/ΔIOP* | -3.1E-05 | 2E-05 | -7E-05 | 8.64E-06 | -1.52864 | 0.13 |
| *E_θθ_/ΔIOP* | 5.67E-06 | 3.17E-05 | -5.6E-05 | 6.78E-05 | 0.178884 | 0.86 |
| *E_rθ_/ΔIOP* | -1.9E-06 | 4.54E-05 | -9.1E-05 | 8.71E-05 | -0.04176 | 0.97 |
| *E_zθ_/ΔIOP* | 5.38E-05 | 0.000114 | -0.00017 | 0.000277 | 0.471598 | 0.64 |
| *E_rz_/ΔIOP* | 3.99E-06 | 1.36E-05 | -2.3E-05 | 3.06E-05 | 0.294515 | 0.77 |
| *E_max_/ΔIOP* | -2.5E-05 | 3.01E-05 | -8.4E-05 | 3.44E-05 | -0.81596 | 0.41 |
| *Γ_max_/ΔIOP* | 1.83E-05 | 3.71E-05 | -5.4E-05 | 9.09E-05 | 0.493472 | 0.62 |
| *ALD/ΔIOP* | 0.014026 | 0.023577 | -0.03218 | 0.060237 | 0.5949 | 0.55 |

Supplemental Table 21: RED RNFL subgroup: GEE of the effects of MD on LC compliance (or ALD compliance) with effects for left and right eyes from the same patient for glaucoma eyes with red RNFL measurements.

|  | Estimate | Standard error | Lower confidence Limits | Upper confidence Limits | Z | *p*-value |
| --- | --- | --- | --- | --- | --- | --- |
| *E_zz_/ΔIOP* | -7.7E-05 | 1.64E-05 | -0.00011 | -4.5E-05 | -4.67435 | **<0.0001** |
| *E_rr_/ΔIOP* | 1.81E-06 | 2.82E-05 | -5.3E-05 | 5.71E-05 | 0.064137 | 0.95 |
| *E_θθ_/ΔIOP* | -1.9E-05 | 3.39E-05 | -8.5E-05 | 4.78E-05 | -0.5528 | 0.58 |
| *E_rθ_/ΔIOP* | 4.64E-05 | 3.81E-05 | -2.8E-05 | 0.000121 | 1.219069 | 0.22 |
| *E_zθ_/ΔIOP* | -0.00022 | 9.44E-05 | -0.0004 | -3.4E-05 | -2.32397 | **0.02** |
| *E_rz_/ΔIOP* | -3.3E-05 | 3.17E-05 | -9.5E-05 | 2.96E-05 | -1.0267 | 0.30 |
| *E_max_/ΔIOP* | -4E-05 | 6.21E-05 | -0.00016 | 8.18E-05 | -0.64306 | 0.52 |
| *Γ_max_/ΔIOP* | -8E-05 | 6.25E-05 | -0.0002 | 4.25E-05 | -1.27996 | 0.20 |
| *ALD/ΔIOP* | 0.041451 | 0.031624 | -0.02053 | 0.103433 | 1.310734 | 0.19 |

Supplemental Table 22: RED RNFL subgroup: GEE of the effects of VFI on LC compliance (or ALD compliance) with effects for left and right eyes from the same patient for glaucoma eyes with red RNFL measurements.

|  | Estimate | Standard error | Lower confidence Limits | Upper confidence Limits | Z | *p*-value |
| --- | --- | --- | --- | --- | --- | --- |
| *E_zz_/ΔIOP* | -2.3E-05 | 2.52E-07 | -2.4E-05 | -2.3E-05 | -91.4647 | **<0.0001** |
| *E_rr_/ΔIOP* | -1.2E-06 | 9.92E-06 | -2.1E-05 | 1.82E-05 | -0.12109 | 0.90 |
| *E_θθ_/ΔIOP* | -1.7E-05 | 1.37E-05 | -4.3E-05 | 1.01E-05 | -1.21869 | 0.22 |
| *E_rθ_/ΔIOP* | 1.47E-05 | 1.15E-05 | -7.9E-06 | 3.74E-05 | 1.275057 | 0.20 |
| *E_zθ_/ΔIOP* | -8.4E-05 | 2.82E-05 | -0.00014 | -2.9E-05 | -2.97795 | **0.002** |
| *E_rz_/ΔIOP* | -1.3E-05 | 1.1E-05 | -3.5E-05 | 8.19E-06 | -1.214 | 0.22 |
| *E_max_/ΔIOP* | -1E-05 | 1.63E-05 | -4.2E-05 | 2.18E-05 | -0.61836 | 0.54 |
| *Γ_max_/ΔIOP* | -2.5E-05 | 1.82E-05 | -6.1E-05 | 1.06E-05 | -1.3756 | 0.17 |
| *ALD/ΔIOP* | 0.004651 | 0.010454 | -0.01584 | 0.025141 | 0.444915 | 0.66 |

Supplemental Table 23: RED RNFL subgroup: Multivariate GEE of the effects of IOP increase and Age on LC strain with effects for left and right eyes from the same patient for glaucoma eyes with red RNFL measurements.

DV = dependent variable, IV = Independent Variable

| DV | Estimate | Standard error | Lower confidence Limits | Upper confidence Limits | Z | *p*-value | IV |
| --- | --- | --- | --- | --- | --- | --- | --- |
| *E_zz_* | 0.000241 | 0.000255 | -0.00026 | 0.00074 | 0.945764 | 0.34 | IOP |
| *E_zz_* | 0.00015 | 6.1E-05 | 3E-05 | 0.000269 | 2.451025 | **0.01** | Age |
| *E_rr_* | -9E-06 | 0.000137 | -0.00028 | 0.000259 | -0.06574 | 0.95 | IOP |
| *E_rr_* | -4E-05 | 5.56E-05 | -0.00015 | 6.87E-05 | -0.72509 | 0.47 | Age |
| *E_θθ_* | -0.00028 | 0.000287 | -0.00085 | 0.000278 | -0.99115 | 0.32 | IOP |
| *E_θθ_* | -5.2E-06 | 0.000101 | -0.0002 | 0.000193 | -0.05167 | 0.96 | Age |
| *E_rθ_* | 0.000241 | 0.000138 | -2.9E-05 | 0.000512 | 1.747484 | 0.080 | IOP |
| *E_rθ_* | 4.16E-05 | 8.59E-05 | -0.00013 | 0.00021 | 0.484768 | 0.63 | Age |
| *E_zθ_* | -7.7E-05 | 0.000259 | -0.00059 | 0.000431 | -0.29821 | 0.77 | IOP |
| *E_zθ_* | -0.00033 | 0.000123 | -0.00057 | -9.2E-05 | -2.70662 | **0.007** | Age |
| *E_rz_* | 3.23E-05 | 0.000156 | -0.00027 | 0.000338 | 0.206842 | 0.84 | IOP |
| *E_rz_* | -2.2E-05 | 6.09E-05 | -0.00014 | 9.72E-05 | -0.36422 | 0.72 | Age |
| *E_max_* | 0.000409 | 0.000336 | -0.00025 | 0.001067 | 1.21845 | 0.22 | IOP |
| *E_max_* | -1.7E-05 | 6.83E-05 | -0.00015 | 0.000117 | -0.25017 | 0.80 | Age |
| *Γ_max_* | 0.000293 | 0.000224 | -0.00015 | 0.000733 | 1.308181 | 0.19 | IOP |
| *Γ_max_* | -7.2E-05 | 5.61E-05 | -0.00018 | 3.78E-05 | -1.286 | 0.20 | Age |
| *ALD* | -0.61229 | 0.249264 | -1.10084 | -0.12374 | -2.4564 | 0.014 | IOP |
| *ALD* | -0.03537 | 0.053773 | -0.14077 | 0.070019 | -0.65784 | 0.51 | Age |

Supplemental Table 24: RED RNFL subgroup: GEE of the effects of Age on LC compliance (or ALD compliance) with effects for left and right eyes from the same patient for glaucoma eyes with red RNFL measurements.

|  | Estimate | Standard error | Lower confidence Limits | Upper confidence Limits | Z | *p*-value |
| --- | --- | --- | --- | --- | --- | --- |
| *E_zz_/ΔIOP* | 2.8E-05 | 1.3E-05 | 2.47E-06 | 5.35E-05 | 2.149559 | **0.03** |
| *E_rr_/ΔIOP* | -8.9E-06 | 7.38E-06 | -2.3E-05 | 5.61E-06 | -1.19994 | 0.23 |
| *E_θθ_/ΔIOP* | -7.9E-06 | 1.32E-05 | -3.4E-05 | 1.8E-05 | -0.59995 | 0.55 |
| *E_rθ_/ΔIOP* | 8.32E-06 | 1.15E-05 | -1.4E-05 | 3.09E-05 | 0.721345 | 0.47 |
| *E_zθ_/ΔIOP* | -6.3E-05 | 1.56E-05 | -9.3E-05 | -3.2E-05 | -4.04748 | **<0.0001** |
| *E_rz_/ΔIOP* | -1E-05 | 9.59E-06 | -2.9E-05 | 8.65E-06 | -1.0584 | 0.29 |
| *E_max_/ΔIOP* | -1.5E-05 | 1.41E-05 | -4.2E-05 | 1.28E-05 | -1.05136 | 0.29 |
| *Γ_max_/ΔIOP* | -2.7E-05 | 1.86E-05 | -6.3E-05 | 9.99E-06 | -1.42383 | 0.15 |
| *ALD/ΔIOP* | -0.01233 | 0.006994 | -0.02604 | 0.001376 | -1.76325 | 0.078 |
